# Remodeling of the glutamatergic system in the Alzheimer’s disease medial temporal lobe and superior temporal gyrus

**DOI:** 10.4103/NRR.NRR-D-25-01177

**Published:** 2026-01-02

**Authors:** Marina Wasef, Andrea Kwakowsky

**Affiliations:** Pharmacology and Therapeutics, School of Pharmacy and Medical Sciences, Galway Neuroscience Center, University of Galway, Galway, Ireland

Alzheimer’s disease (AD) is a progressive neurodegenerative disease characterized by memory loss and cognitive decline. Beyond the accumulation of amyloid-beta (Aβ) plaques and tau neurofibrillary tangles, the hallmark pathologies of AD, glutamatergic dysregulation, and excitotoxicity are key contributors to disease progression. Given that glutamate is the main excitatory neurotransmitter and has a central role in the regulation of synaptic plasticity, memory, and learning, any disturbance in glutamate signaling can disrupt normal brain function (Yeung et al., 2021a; Soares et al., 2024). The medial temporal lobe and superior temporal gyrus (STG) are severely impacted in AD, and the remodeling of glutamate receptors and transporters has been extensively examined as mechanisms linked to neuronal network dysfunction (Yeung et al., 2021a, b; Soares et al., 2024; Wood et al., 2024).

Glutamate acts on ionotropic and metabotropic receptor families. Ionotropic glutamate receptors are primarily categorized into three types: N-methyl-D-aspartate receptors (NMDAR), AMPA (alpha-amino-3-hydroxy-5-methyl-4-isoxazolepropionic acid) receptors (AMPAR), and kainate (GluK) receptors. The receptors are formed by multiple subunits, which are classified as follows: GluN1-3 (NMDA), GluA1-4 (AMPA), GluK1-5 (kainate), and mGluR1-5 (metabotropic). The control of synaptic vesicle filling with glutamate for release is mediated by the vesicular glutamate transporters (VGLUTs). VGLUT1 and VGLUT2 are the most common and highly expressed subtypes in the medial temporal lobe and STG (Wood et al., 2024). Glutamate levels in the synaptic cleft are tightly controlled by a range of transporters and enzymes. These include the five excitatory amino acid transporters (EAATs), of which the EAAT2 isoform performs 90% of glutamate reuptake from the synaptic cleft, with a significant role in pathological processes, including in AD (Wood et al., 2022). While EAAT1 has also been linked with disease pathology, its involvement is less studied (Wood et al., 2025). In our recent studies, we characterized the expression of glutamatergic signaling components, such as several glutamate receptor subunits, VGLUTs, EAAT1, and EAAT2, in the human medial temporal lobe and STG in AD (Yeung et al., 2021a, b, 2022; Wood et al., 2024, 2025).

**Figure 1:**
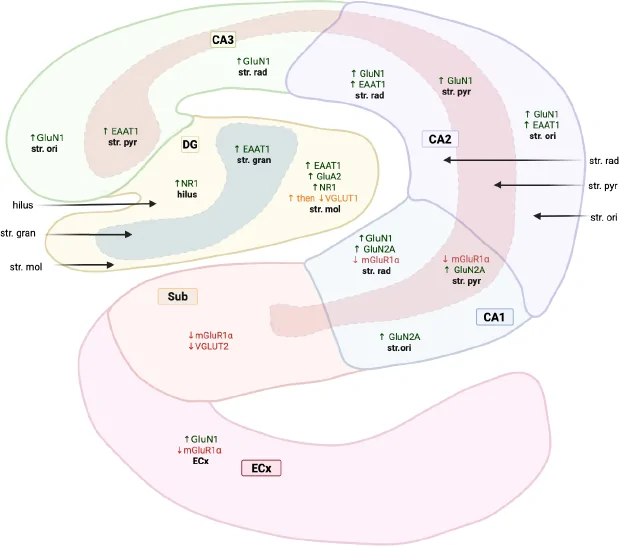
Expression changes of glutamatergic signaling components in the human Alzheimer’s disease hippocampal formation. Created with BioRender.com. CA: Cornus ammonis; DG: dentate gyrus; ECx: entorhinal cortex; str. gran: stratum granulosum; str. mol.: stratum moleculare; str. ori: stratum oriens; str. pyr: stratum pyramidale; str. rad: stratum radiatum; Sub: subiculum.

Aβ impairs synaptic glutamate clearance, causing NMDAR overstimulation and downstream calcium signaling disruptions (Yeung et al., 2021a). This increased activity is in line with our recent findings of significantly increased NMDAR subunit expression (**Figure 1**), including GluN2A levels in the stratum oriens, pyramidale, and radiatum of the CA1 subregion, and a trend towards an increase in CA2-3, dentate gyrus (DG), subiculum (Sub), entorhinal cortex (ECx), and STG (Yeung et al., 2021a). Similarly, GluN1 receptor expression is elevated in the DG stratum moleculare and hilus, CA2/CA3 stratum oriens, CA2 stratum pyramidale, CA1–3 stratum radiatum, and ECx neuropil (Yeung et al., 2021a). Conversely, overall perineuronal GluN1 density is paradoxically diminished in the hippocampus, Sub, ECx, and STG, potentially reflecting subregional vulnerability or compensatory astrocytic mechanisms (Yeung et al., 2021a). No significant difference in GluN1 subunit density in the parahippocampal gyrus, frontal lobe, Sub, and STG was observed between AD and controls (Yeung et al., 2021a; **Figure 1**).

NMDARs modulate the activity of protein interacting with C kinase 1 through calcium influx, influencing downstream AMPARs. In a mouse study, both Aβ and tau proteins were shown to interfere with this regulation, increasing GluA2 subunit expression, thereby disrupting AMPAR endocytosis and synaptic localization (Yeung et al., 2021a). In vulnerable hippocampal sectors in AD, including the CA1, Sub, and ECx, we discovered a trend towards decreased GluA2 subunit expression, whereas more resistant regions, such as the stratum moleculare of the DG, have marked increases in GluA2 expression, likely a protective adaptation. Namely, increased GluA2 expression could limit cation influx, suppressing downstream excitotoxic NMDAR activation (Yeung et al., 2021a). We also found unchanged GluA2 subunit expression in the ECx and STG (**Figure 1**).

mGLUR alterations were also explored in the AD brain. mGluRs are G-protein coupled receptors which have a wide-ranging functionality, including the regulation of neuronal injury and survival. Group I mGluRs (mGluR1 and mGluR5) are linked with ionotropic receptor activation. The mGluR subtype 1 splice variant (mGluR1α) is the longest variant of the mGluR1 receptor and is linked to AD pathology. Aβ may contribute to pyramidal cell hyperactivity through mGluR1 activation, facilitating hippocampal microglial-mediated glutamatergic synaptic phagocytosis (Yeung et al., 2022). In the human AD brain, we found that the Sub and ECx had significantly decreased expression in mGluR1α (Yeung et al., 2022). We also observed markedly reduced diffuse staining and weaker immunoreactivity in the CA1 stratum pyramidale and radiatum, whereas the stratum oriens appears unaffected (Yeung et al., 2022; **Figure 1**). Group I mGluRs within the frontal cortex in Lewy Body Dementia and AD have also demonstrated a decrease in expression of mGluR1 correlating with AD severity, but no significant changes in mGluR5 were observed (Yeung et al., 2022).

mGluR1α is localized to perisynaptic zones, making it essential for glutamate regulation. Excess glutamate or altered glutamate reuptake would cause glutamate to diffuse to the perisynaptic regions (Yeung et al., 2022). The excess glutamate due to downregulated mGluR1a, as observed in our studies, may promote excitotoxicity through sustained receptor activation and excessive calcium signaling (Yeung et al., 2022). Alternatively, the excess glutamate may activate the remaining mGluR1α to promote Group I mGluR-mediated AMPAR endocytosis, reduce NMDAR activity, and avoid excitotoxic effects (Yeung et al., 2022). The understanding of the complex interplay between these different types of glutamatergic receptors is far from complete and requires elucidating the mechanisms of synapse dysregulation in AD.

Presynaptic glutamate transport is affected in AD. VGLUT1 and VGLUT2, which are essential for packaging glutamate into synaptic vesicles, are lost in the frontal, parietal, and occipital cortices (Wood et al., 2024). Additionally, VGLUT1 is decreased in the DG, and VGLUT2 in the Sub and STG in AD (Wood et al., 2024). A previous study suggests that VGLUTs are preferentially co-expressed with Aß, pointing to Aß-associated glutamate transport dysfunction (Wood et al., 2024). This contributes to elevated intracellular glutamate concentration, inducing further synaptic dysfunction, increasing release of Aβ from glutamatergic synapses (Wood et al., 2024). These mechanisms may explain the relative preservation of VGLUT levels in the medial and temporal regions, as amyloid plaque accumulation in these areas happens after other regions (Wood et al., 2024). However, no correlation between VGLUT levels and amyloid plaques was observed in our studies, suggesting other pathological mechanisms may be triggering the reduced transporter expression (Wood et al., 2024; **Figure 1**).

Another potential mechanism, as pointed out by Siano et al. (2020), is the possible modulation of VGLUT1 expression by tau protein through this protein’s translocation to the nucleus. In the early stages of AD in a mouse study, tau detachment from microtubules and translocation to the nucleus upregulated VGLUT1 hippocampal expression, resulting in increased glutamate release and ultimate hyperexcitability (Siano et al., 2020). However, as the disease progresses, tau aggregation into insoluble tangles results in loss of its regulatory function, causing VGLUT1 expression reduction, resulting in decreased glutamate release and subsequent hypo-excitation (Siano et al., 2020). Thus, tau pathology may cause a biphasic effect on VGLUT1 expression: enhancing it in early stages, then dampening it in later stages (Wood et al., 2024).

Unlike VGLUT1 density decreases in both the outer and inner molecular layer of the DG, presynaptic vesicle fusion protein components loss is restricted to the outer layer, indicating differential regulation (Wood et al., 2024). Nonetheless, overall, there is an ultimate decrease in glutamate release, which may be a protective mechanism to reduce glutamate excitatory transmission and excitotoxic damage in the DG (Wood et al., 2024). Furthermore, a decreased vesicle fusion protein synaptophysin and increased VGLUT2 levels in the ECx are reported, suggesting competing glutamate release dynamics (Wood et al., 2024). These contradictory findings result in unclear effects on overall glutamate release, underscoring the need to further investigate the distinctive regulation of VGLUT and vesicle fusion proteins in AD. Furthermore, we found a trend towards decreased VGLUT2 density in the ECx in AD (**Figure 1**).

We also discovered that VGLUT2 expression is preserved in the hippocampus but is significantly reduced in the Sub and STG (Wood et al., 2024). VGLUT2 is a marker for burst firing subicular output neurons in the hippocampus (Wood et al., 2024). Two types of subicular output neurons include burst-firing and regular-firing neurons. Regular firing neurons, which represent most neurons and are mainly located in the proximal subiculum, fire one action potential when depolarized. In contrast, burst-firing neurons, which are in the distal subiculum and project to medial ECx, retrosplenial cortex, and hypothalamus, fire multiple action potentials when depolarized. VGLUT2 density down-regulation reflects lower expression on burst-firing neurons, suggesting altered hippocampal-cortical connectivity and circuit disruption (Wood et al., 2024).

We also explored the expression of EAATs in the AD medial temporal lobe, ECx, and STG. These glutamate transporters are primarily astrocytic and are important in maintaining glutamate homeostasis by clearing excess glutamate from the synapse (Yeung et al., 2021b). Each EAAT transport cycle consists of co-transportation of one glutamate molecule with three Na^+^ and one H^+^ against one K^+^ (Yeung et al., 2021b). Due to its high expression in the brain and importance in glutamate clearance, EAAT2 attracted a lot of attention in AD research. We found no significant alterations in EAAT2 density in the AD hippocampus, Sub, ECx, or STG, but did observe intensified, diffuse staining, mainly surrounding astrocytic processes, as shown by immunohistochemistry (Yeung et al., 2021b). The widespread labeling suggests a redistribution of EAAT2 or a compensatory response to elevated extracellular glutamate levels (Yeung et al., 2021b). Notably, while individual astrocytes are not outlined, overall transporter density appears unchanged, potentially due to increased neuropil expression in AD (Yeung et al., 2021b). Additionally, Sasaki et al. (2009) reported EAAT2 co-precipitate with phosphorylated tau, and Hoshi et al. (2018) described its co-expression with aquaporin-4, indicating a possible loss of membrane-bound EAAT2 and formation of insoluble aggregates (Wood et al., 2022). These aggregates may also account for the diffuse EAAT2 immunolabeling observed in our lab (Wood et al., 2022). Further research will need to determine if, in the AD brain, EAAT2 is lost on astrocytic membranes or EAAT2 labeling becomes more pronounced on fine astrocytic processes.

Prior studies report conflicting findings regarding overall EAAT2 levels in AD (Wood et al., 2022; Soares et al., 2024). A study observed decreased EAAT2 protein expression despite unchanged mRNA levels, suggesting post-translational disruption (Wood et al., 2022). Others attempted to reconcile EAAT2 expression discrepancies by noting inter-patient variability and a significant inverse relationship between GFAP and EAAT2 expression (Wood et al., 2022). These differences need to be further researched to explore whether transporter expression is associated with clinical symptoms and with case characteristics.

EAAT2 deficiency is more pronounced in astrocytes than neurons and aligns with AD-associated gene expression patterns (Wood et al., 2022). Although neuronal EAAT2 represents only 20% of total EAAT2, it may account for a greater proportion of glutamate uptake in AD, potentially due to astrocytic EAAT2 mislocalization or inactivation (Wood et al., 2022). A previous study also underscores the possible technical complications of common tissue handling in underestimating astrocytic EAAT2 function (Wood et al., 2022). The exact diﬀerences in physiological function between neuronal and astrocytic EAAT2 are still not well-understood; therefore, their contribution to AD pathogenesis is also unclear.

Despite its neglected less-researched past, EAAT1, another astrocytic transporter, shows a consistent upregulation in AD hippocampus. We observed significantly elevated EAAT1 density in the CA2 stratum oriens and stratum radiatum, the CA3 stratum pyramidale, and in both the stratum moleculare and stratum granulosum of the DG (Wood et al., 2025). A trend toward increased expression was also noted in the CA1 stratum pyramidale and CA3 stratum oriens (Wood et al., 2025; **Figure 1**).

A previous study supports regional variability in EAAT1 expression. Earlier reports found no change in EAAT1 expression in the temporal cortex and cingulate gyrus in AD but did report increased expression in layers III and IV of temporal cortex pyramidal neurons (Wood et al., 2025). This upregulation was co-localized with tau pathology, suggesting a possible association between tau accumulation and elevated EAAT1 expression (Wood et al., 2025). Although most EAAT1-positive cells in the AD brain co-localize with tau, a subset does not, suggesting that the association between EAAT1 expression and tau pathology is not entirely causal (Wood et al., 2025). Given the extensive tau pathology in the AD hippocampus, EAAT1 upregulation may be related to tau but is likely influenced by other contributing mechanisms (Wood et al., 2025). Conversely, we observed no significant EAAT1 density alteration in the STG, which could relate to stage-dependent spread of neurofibrillary and tau tangles (Wood et al., 2025). In earlier disease stages, the STG remains unaffected by tau pathology, with notable tau accumulation generally emerging only at more advanced Braak stages (V–VI) (Wood et al., 2025). Our cohort, with primarily Braak stage IV cases, aligns with this interpretation (Wood et al., 2025). In contrast, the hippocampus, affected earlier in disease progression, exhibited elevated EAAT1 density (Wood et al., 2025). High EAAT1 density may be a compensatory response to increase clearance of a more prominent glutamate release in the hippocampus compared to the STG (Wood et al., 2025). However, this hypothesis warrants further investigation. AD cases in this cohort did not show evident correlation of transporter density to Braak and Braak stage, or tau loads (Wood et al., 2025).

Technical misinterpretations have also influenced EAAT1 localization studies. Previous studies described EAAT1 labeling as neuron-like due to the absence of neuronal markers, which may have led to the misidentification of astrocytic processes as neurons (Wood et al., 2025). In our analysis, we employed GFAP as an astrocyte marker and NeuN as a neuronal marker (Wood et al., 2025). The EAAT1-positive cells were GFAP-positive and NeuN-negative, indicating that the expression was astrocytic rather than neuronal (Wood et al., 2025). Our high-resolution imaging revealed that EAAT1 was predominantly localized to the neuropil in both AD and control brains, with increased immunoreactivity in AD that was closely associated with astrocytic processes (Wood et al., 2025). This elevation may reflect either an upregulation of the transporter itself or an increase in astrocytic density (Wood et al., 2025). As the antibody used targets a C-terminal epitope, it cannot distinguish between full-length and spliced EAAT1 isoforms, making it unclear whether alternative variants contribute to the observed changes (Wood et al., 2025).

In conclusion, glutamatergic dysfunction plays a key role in the progression of AD through glutamate receptor overactivation, dysfunction, and impaired transmitter release and reuptake, which are also linked to changes at the protein expression level. The differences seen across brain regions in NMDA and AMPA receptor subunits, along with the altered patterns of VGLUT and EAAT expression, reflect the complexity of glutamate regulation as the disease progresses. Rather than a uniform decline, glutamatergic changes appear to reflect local compensations or vulnerabilities in response to pathological stressors such as tau or amyloid buildup. Understanding these region-specific patterns of the disturbed glutamatergic homeostasis may help guide more targeted therapeutic approaches aimed at restoring glutamate balance and protecting vulnerable neural circuits in AD.
